# Healthcare reimbursement costs of children with type 1 diabetes in the Netherlands, a observational nationwide study (Young Dudes-4)

**DOI:** 10.1186/s12902-018-0287-6

**Published:** 2018-08-17

**Authors:** E. A. J. M. Spaans, P. R. van Dijk, K. H. Groenier, P. L. P. Brand, N. Kleefstra, H. J. G. Bilo

**Affiliations:** 1 Diabetes Centre, Isala, P.O. box 10400, 8000 GK Zwolle, the Netherlands; 2 Princess Amalia Children’s Clinic, Isala, Zwolle, the Netherlands; 3 Department of Internal Medicine, Isala, Zwolle, the Netherlands; 4 Department of Internal Medicine, University of Groningen and University Medical Center Groningen, Groningen, the Netherlands; 5 Department of General Practice, University of Groningen and University Medical Center Groningen, Groningen, the Netherlands; 6 Langerhans Medical Research Group, Zwolle, the Netherlands; 7 UMCG Postgraduate School of Medicine, University Medical Center and University of Groningen, Groningen, the Netherlands

**Keywords:** Diabetes mellitus, Hospital admission, Children, Reimbursement costs, Nationwide

## Abstract

**Background:**

Type 1 diabetes mellitus (T1DM) is one of the most common chronic diseases in children. Studies on costs related to T1DM are scarce and focused primarily on the costs directly related to diabetes. We aimed to investigate both the overall healthcare costs and the more specific costs related to the management of diabetes.

**Methods:**

This is a retrospective and observational, nationwide cohort study of all Dutch children (aged 0–18 years) with T1DM. Data were collected from the national registry for healthcare reimbursement, in which all Dutch insurance companies combine their reimbursement data. In the Netherlands for all Dutch citizens health care is covered by law and all children are treated by hospital-based paediatricians.

**Results:**

We analysed 6710 children distributed over 81 hospitals: 475 children in 6 university hospitals and 6235 children in 75 general hospitals. Total reimbursement for all children with T1DM over the period 2009 to 2011 was € 167,494,732 corresponding to an annual mean of € 55,831,577 of total costs and € 8326 euros per child. When comparing small (between 26 and 54 patients), medium (57–84 patients) and large (88–248 patients) general hospitals, costs per patient were highest in the hospitals with the highest number of T1DM patients. The costs for devices, secondary care and pharmaceutics had most impact on total expenditures. Over the study period, there was a slight decrease in per person costs.

**Conclusion:**

The overall health expenditure of a child with T1DM is more than € 8000 per patient per annum. Given the move towards more device-intensive multidisciplinary care for these patients, the costs of treating T1DM in children are likely to increase further in the coming years.

## Background

Type 1 diabetes mellitus (T1DM) is one of the most common chronic diseases in children in developed nations [1]. The incidence rate of T1DM in the Netherlands has doubled over the past three decades to 21 per 100,000 children aged 14 years and younger [2]. The management of T1DM is intensive and complex. During the past decades, T1DM management has evolved from a physician-patient relation using ‘one-size fits all’ multiple daily injection insulin therapy to a multidisciplinary team approach with new insulin preparations, insulin pump therapy, and (continuous) glucose sensors. It is likely that this move towards more complex, intensive and multidisciplinary care raises the costs of treatment per child. Together with the increase in prevalence rates of T1DM in children this will have considerable impact on the budget needed to deliver appropriate care for these patients. More insight in such changes allows for better planning and a more solid corroboration of the needed funds.

There are currently only a few studies reporting the costs of T1DM among children [3–6]. An increase in the costs over the last decades was observed by most of these studies [4, 7]. These studies reported that hospital (re-)admissions, devices and medication are the bulk of healthcare expenses. There is also an indication that the number of re-admissions is lower in larger hospitals [8]. However, most of these reports were of a cross-sectional nature. Furthermore and importantly, as these studies only reported the costs directly related to diabetes, the overall healthcare costs for children with T1DM remain unknown. When assessing information with regard to total health care expenditure for adult persons with diabetes, data suggest that more expenditure is needed for general care not directly related to diabetes compared to diabetes related care [9]. To our knowledge, a comparable analysis has not been performed in children with T1DM.

The aim of the present study was to investigate both the overall and diabetes-specific healthcare costs as derived from reimbursement data related to the management of Dutch children with T1DM aged 0 to 18 years. In order to gain more insight in the various aspects of costs, we also investigated its course, determinants and differences according to size of hospitals.

## Methods

### Study design

Retrospective, observational, nationwide study in the Netherlands study covering a period of 3 years (2009 to 2011). This study is part of the Young DUtch Diabetes Estimates (DUDE) initiative, a nation-wide project aimed at investigating the magnitude and impact of diabetes mellitus, complications and costs among children and adolescents in the Netherlands [2]. Aim of the present analysis was to investigate the overall and diabetes-specific healthcare costs related to the management of Dutch children (aged 0 to 18 years) with T1DM.

### Outcomes

Primary outcome measure was the annual total healthcare costs related to children with T1DM (aged 0 to 18 years) in the period 2009 to 2011 in the Netherlands. As a secondary outcome measure we divided the costs according to nature (pharmaceuticals, device related, primary and secondary care consultations, admissions, maternity care, dental health and mental health). We also analysed the following putative determinants of costs: hospital type (university hospitals and general hospitals divided into tertiles according to the number of T1DM patients cared for in the hospital; hospitals caring for less than 20 children with T1DM were excluded from analysis) and number of readmissions.

The available data on reimbursed health care expenditure were not corrected for Consumer Price Index (CPI) changes. CPI changes are known for the Netherlands, being 1.3% for 2010 vs 2009, and 2.3% for 2011 vs 2010. However, in the Netherlands, not all expenditure is included in the calculation of the CPI: income tax, social premiums and spending on insured health care, for example, are nor taken into account (https://www.cbs.nl/en-gb/background/2005/26/consumer-price-index). Therefore, correcting for the CPI would not add to a better understanding of the available information.

### Data collection

Throughout the 3-year study period, reimbursement of all hospital care was handled through the registration as Diagnosis Treatment Combination (Diagnose-Behandel Combinatie (DBC) in Dutch) codes. All Dutch children with T1DM are treated by hospital-based paediatricians and these physicians are required to record information by the appropriate DBC codes [2]. Importantly, each DBC code contains information about the attending physician (e.g. the specific specialty), the diagnosis, and the type of care provided. All DBC codes are stored in a national database, managed by Vektis (Vektis, Zeist, The Netherlands; https://www.vektis.nl/). Besides this database Vektis also manages other databases including the Basic Health Insurance Information System. This database contains demographic information (e.g. date of birth and gender) and information on drug prescription, for all children that are registered as inhabitants in the Netherlands. The coverage of this system is approximately 98% [10]. Claims records for pharmaceutical care, with a coverage of 99%, were derived from the Pharmacy Information System, containing information on the date the drug was supplied, the physician prescribing the drug, the specific drug that was supplied (including Anatomical Therapeutic Chemical (ATC) code), and the quantity supplied. Since all healthcare system records, including the Pharmacy Information System, use the same unique identifying number for each patient (the ‘Citizen Service Number’), we were able to link all claims for any individual together and thereby track each individual through all domains of healthcare and over time [11].

Resource costs were derived from the BASIC Detail Information database. This database provides insight into the total reimbursement of declared DBCs and other items as described below, under the Health Insurance Act (which then can be seen as a rather accurate proxy of costs of care), classified by type of healthcare procedure and aggregated into specific cost categories. The specific categories are: primary care, medications, dental care, obstetric care, paramedical assistance (including physiotherapy, speech therapy practice, dietetics), devices, patient transport and travel related costs, maternity care, mental health, and secondary care. Coverage of the BASIC Detail Information database is approximately 100%. Data available for research purposes were stripped from identifying characteristics to ensure anonymity: the Citizen Service Number was encrypted, date of birth converted into the person’s age, and the postal code recoded to limit its identifying properties to hospital level. All hospitals were numbered to avoid direct recognition of specific hospitals. We extracted data about overall health care expenditure and more detailed in accordance with the above named subdivision.

### Patients

We selected children aged 18 years or younger on the 1st of July for every single year of the study period (2009–2011). In this group, children with at least one DBC claim for diabetes mellitus (paediatrics code [0316] and diabetes diagnosis code [7104], or internal medicine code [0313] and diabetes diagnosis code [221, 222 or 223]) were included.

### Statistical analysis and ethical considerations

Statistical analyses were carried out using SPSS (IBM SPSS Statistics for Windows, Version 20.0. Armonk, NY: IBM Corp.). All costs are expressed in Euros (€). As retrospective studies using anonymized data are exempt from ethical review under Dutch law, medical ethics approval was not required for this study. This was confirmed in writing by the ethical review board of Isala hospital.

## Results

A total of 91 hospitals (8 university and 83 general hospitals) were included. Two university hospitals and 8 general hospitals were excluded because they cared for less than 20 children with T1DM each. The study sample comprised 6710 children whose T1DM was managed at 81 hospitals: 475 children in 6 university hospitals and 6235 children in 75 general hospitals. The number of children per year is presented in Table 1.

**Table 1:** Costs (in euros) for management of type 1 diabetes in the period 2009 to 2011 in the Netherlands

	2009	2010	2011	Total	Mean
General hospitals					
Total	52,936,879	53,273,011	49,431,443	155,641,333	51,880,444
Mean	8639	8570	7769	24,977	8326
Number of children	6128	6216	6363	18,707	6236
Large					
Total	33,420,336	33,039,872	31,393,730	97,853,938	32,617,979
Mean	9382	9087	8277	26,746	8915
Number of children	3562	3636	3793	10,991	3664
Medium					
Total	11,917,983	12,481,770	11,491,543	35,891,295	11,963,765
Mean	7496	7681	7107	22,283	7428
Number of children	1590	1625	1617	4832	1611
Small					
Total	7,598,559	7,751,370	6,546,171	21,896,100	7,298,700
Mean	7785	8117	6869	22,771	7590
Number of children	976	955	953	2884	961
University hospital					
Total	3,953,900	4,113,517	3,785,982	11,853,399	3,951,133
Mean	8036	9223	7790	25,050	8350
Number of children	492	446	486	1424	475
Total					
Total	56,890,779	57,386,528	53,217,426	167,494,732	55,831,577
Mean	8594	8614	7770	24,978	8326
Number of children	6620	6662	6849	20,131	6710

Costs are subdivided according to type and volume of hospital; and are presented as total costs (upper row) and mean costs per child (middle row). The total number of children is presented in the lower row

The total reimbursement for all children with T1DM over the period 2009 to 2011 was € 167,494,732 (Table 1), corresponding to a mean of € 8326 per child per year. Mean costs were € 8326 per child when children were seen in general hospitals for their T1DM, and € 8350 when children were seen in a university hospital. Costs for devices, medical specialist consultations and pharmaceutics had most impact on the total expenditures (Table 2). Over the study period, there was a decrease in costs, both for general and university hospitals. In general, the costs for general practitioners and paramedical care increased while costs for mental health, dental health and secondary care decreased.

**Table 2:** Determinants of the costs (in euros) for management of type 1 diabetes in the period 2009 to 2011 in the Netherlands

	2009	2010	2011	Mean
Dental health				
Total	847,398	919,940	824,414	863,917
Mean	128	138	120	129
Device-related costs				
Total	22,169,761	22,806,472	22,383,567	22,453,267
Mean	3349	3423	3268	3347
First-line care				
Total	780,656	753,617	852,229	795,500
Mean	59	57	62	59
Maternitiy care				
Total	5500	2008	2402	3304
Mean	0	0	0	0
Secundary care				
Total	22,495,905	21,743,263	18,468,504	20,902,557
Mean	3398	3264	2697	3119
Mental health				
Total	3,232,896	3,482,130	2,777,693	3,164,239
Mean	488	523	406	472
Other costs				
Total	417,630	409,597	391,223	406,150
Mean	32	31	29	30
Paramedical care				
Total	419,264	486,974	532,576	479,605
Mean	63	73	78	71
Travel related costs				
Total	69,142	67,299	70,042	68.828
Mean	10	10	10	10
Pharmaceuticals				
Total	6,452,628	6,715,226	6,914,777	6,694,210
Mean	975	1008	1010	997
Total				
Total	56.890.779	57.386.528	53.217.426	55.831.577
Mean	8.594	8.614	7.770	8.326

Costs are presented as total costs (upper row) and mean costs per child (lower row)

**Fig. 1:**
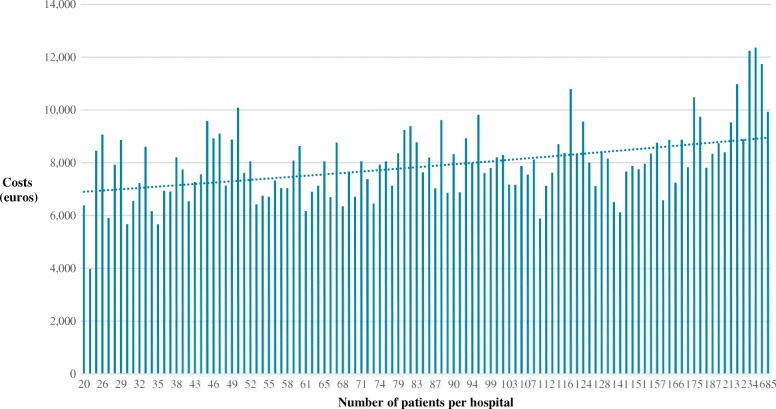
Mean costs (in euros) per child for the management of type 1 diabetes according to the number of patients per hospital

In Fig. 1, The mean costs (in euros) per child according to the number of patients per hospital and the average annual costs (in euros) per child with T1DM are shown when comparing hospitals, caring for different numbers of patients. Costs were highest in hospitals with the largest number of patients (Table 1 and Fig. 1).

## Discussion

This study found that the mean costs of treating T1DM (based on reimbursement data) in children in the Netherlands are € 8326 per child. Healthcare costs associated with children proved to be higher when children were being treated for their diabetes in a larger general hospital, with more or less the same health care expenditure per child as in an university hospital.

The costs of treating a child with T1DM in this study are considerably higher than those reported earlier. In Europe, previous studies from Germany (2007) and Greece (2011 to 2012) reported mean annual costs per child with T1DM of € 3542 and € 2712, respectively [4, 12]. The Greek study included only 89 children [12]. A study from California (2009 to 2012) reported median median annual costs of US$ 7654 (€ 6850 euros) [13], and a small Brazilian study (2008 to 2010) [14], which did not include hospital admissions, reported average annual costs of $ 1319 (€ 1180). Differences in organization and accessibility of health care and standards of diabetes care hamper the comparison of such costs between countries.

Our study is the first to incorporate all costs (including e.g. paramedical and dental care) for children with T1DM. Previous studies only examined costs for drugs, devices or hospital admissions directly associated with diabetes [4, 12, 14]. In addition, differences in the use of devices such as insulin pumps, may have influenced the differences between countries [4, 13, 14]. Apart from hospitalization, the use of such devices is the main determinant of costs for diabetes. The few studies reporting both costs and insulin pump usage showed marked differences in the proportion of T1DM patients using insulin pumps, ranging from 1.2% in Brazil (2008 to 2010) [14], 18% in California (July 1st 2009 to June 30th 2012) [13] to more than 25% in Germany 2007 [8] and 37% in the Netherlands (unpublished data). In addition, studies differ in the proportion of medical costs spent on pharmaceuticals, ranging from 12 to 33%, indicating that differences in the costs of insulin may also influence the results [5, 14]. There are also methodological differences between studies, the different population profiles analyzed and the differences between the unit costs of health services between countries hampering mutual comparison. There also seems to be a relationship between higher costs and poorer metabolic control which is related to increased admissions in children and intensive care [4–6, 8, 15], and a lower socioeconomic status is correlated with higher costs partly as result of a higher admissions rate [8, 16–18]. On the other hand, patients with the poorest control tended to have relatively lower costs for supplies, outpatient visits and laboratory tests but higher hospitalization costs [12].

In the present study, the average annual costs of treating T1DM in children was higher in hospitals caring for more patients (Fig. 1). This is surprising as it is commonly believed that a larger patient volume would allow for lower per person overhead costs, and – thus – to lower overall costs per individual patient. Furthermore, out-of-office hours services are probably better developed in larger institutes. In large centres, the availability of a dedicated 24-h service might allow resolving diabetes specific problems without need for admission. The finding that the number of admissions of children with T1DM is lower in larger centers supports this hypothesis [8, 18]. On the other hand it is likely that larger centers treat more complex cases. Also, other factors such as socio-economic status could be of influence (19). Further studies are needed to identify the factors responsible for the higher costs in larger hospitals. Finally, it should be mentioned that we investigated the sum of all large, medium and small centres and there could be individual centres with lower costs than expected.

Because of the rapid developments in the innovation of the treatment of T1DM [19–22] with an increasing use of high-tech devices, the costs of treating T1DM in children are likely to increase further in the coming years. Data from adults with diabetes suggest that more expenditure is needed for general care not directly related to diabetes compared to diabetes related care [9]. The present study provides insight in the total health care expenditure and specific diabetes-related costs of T1DM care among children in the Netherlands. Hopefully, the insight gained will allow a better understanding and planning of health care needs of this selected population, not only with respect to diabetes, but also in general. The main strength of our study is that we provide a nationwide perspective of all the costs associated with the management of children with T1DM, including dental and mental health. The following limitations should be mentioned. First, we did not examine the influence of patient characteristics on costs. Previous studies found that the cost of girls are higher than for boys [4, 8, 18] and this may by quite different per age category [4, 5, 8]. Second, we were unable to specify the different aspects of the costs on a more detailed level due to the structure of the DBC system in the Netherlands. Finally, the cross-sectional character of our data precludes causal inferences.

## Conclusions

Between 2009 and 2011, the total costs for T1DM in the Netherlands (based on reimbursement data) were more than € 55 million, corresponding to an mean of € 8326 euros per child with T1DM per annum. Costs were highest in hospitals treating a larger numbers of patients and the costs found in the present study seem to be higher than in previous reports from other countries. This may be partly explained by increased device use and differences in healthcare systems and study methods. Given the move towards more device-intensive and multidisciplinary care for children with T1DM, it is likely that the health expenditure for T1DM will continue to rise in the coming years.

## Abbreviations

ATC code: Anatomical Therapeutic Chemical code
CPI: Consumer Price Index
DBC: Dutch Diagnose Behandel Combinatie (diagnosis treatment combination)
DUDEs: DUtch Diabetes Estimates
T1DM: Type 1 diabetes mellitus
